# Supplementary material for: Three Distinct Annotation Platforms Differ in Detection of Antimicrobial Resistance Genes in Long-Read, Short-Read, and Hybrid Sequences Derived from Total Genomic DNA or from Purified Plasmid DNA
Source: Antibiotics (Basel). 2022 Oct 12;11(10):1400. doi: 10.3390/antibiotics11101400 (PMC9598756; doi:10.3390/antibiotics11101400)
Supplement: Supplementary file 1 [file antibiotics-11-01400-s001.zip › Supp table S1_amended.pdf]

Table S1. Concentration and purity of total genomic DNA and pure plasmid DNA preparations.

	E. coli **DU1040 (NR1, control)**	*E. ludwigii* **LST1391B**	*K. pneumoniae* **LST1504-C2**
		Total genomic DNA	
Concentration[a]	184 ng/µL	68 ng/µL	115 ng/µL
A260/280 ratio[b]	1.91	1.81	1.85
A260/230 ratio[b]	2.13	1.86	2.02
		Plasmid DNA	
Concentration[a]	198 ng/µL	127 ng/µL	163 ng/µL
A260/280 ratio[b]	1.82	1.86	1.79
A260/230 ratio[b]	2.02	2.18	1.93

[a]Concentration: measured by Qubit fluorometer using a double-stranded DNA assay kit.
[b]Purity: measure by NanoDrop Spectrophotometer.
