# Supplementary material for: Three Distinct Annotation Platforms Differ in Detection of Antimicrobial Resistance Genes in Long-Read, Short-Read, and Hybrid Sequences Derived from Total Genomic DNA or from Purified Plasmid DNA
Source: Antibiotics (Basel). 2022 Oct 12;11(10):1400. doi: 10.3390/antibiotics11101400 (PMC9598756; doi:10.3390/antibiotics11101400)
Supplement: Supplementary file 1 [file antibiotics-11-01400-s001.zip › Supp table S2_amended.pdf]

Table S2. Unassembled read data from Illumina Iseq and MinION Nanopore sequencing runs.

**Bacterial strain**	**DNA preparation**	**№ of Illumina reads**	**Total base pairs sequenced (Illumina)**	**№ of Nanopore reads**	**Average length of Nanopore reads**
E. coli DU1040 (NR1, control)	total genomic	743,496	53,013,809	333,838	7,600
E. coli DU1040 (NR1, control)	plasmid	959,424	69,593,881	359,687	12,990
E. ludwigii LST1391B	total genomic	1,761,970	127,295,374	18,512	4,699
E. ludwigii LST1391B	plasmid	360,904	25,857,933	46,792	10,139
K. pneumoniae LST1504-C2	total genomic	1,503,146	109,086,227	61,029	7,384
K. pneumoniae LST1504-C2	plasmid	970,470	70,243,877	91,319	6,771
