# Supplementary material for: Three Distinct Annotation Platforms Differ in Detection of Antimicrobial Resistance Genes in Long-Read, Short-Read, and Hybrid Sequences Derived from Total Genomic DNA or from Purified Plasmid DNA
Source: Antibiotics (Basel). 2022 Oct 12;11(10):1400. doi: 10.3390/antibiotics11101400 (PMC9598756; doi:10.3390/antibiotics11101400)
Supplement: Supplementary file 1 [file antibiotics-11-01400-s001.zip › Supp table S5_amended.pdf]

Table S5. Plasmid taxonomic classification by COPLA.

Strain	Known or estimated size	Plasmid taxonomic classification				
		**PTU**[a]	**Inc groups**[b]	**MOB**[c]	**MPF**[d]	**Conjugation genes**
E. coli DU1040 (NR1) - control	94	PTU-FE III	IncFII	MOBF	Type F	*virb4, MOBB, t4cp1 traF, traG, traN, trbC, traH, traW, traU*
K. pneumoniae LST1504-C2	~232	PTU-HI1B	IncFIB(Mar)	MOBH	-	-
	~153	NA	IncFIB(K)	MOBF	-	-
	~71	NA	IncFII(Yp)	-	-	-
	~6	PTU-E71III	NA	-	Col440I	-
E. ludiwigii LST1391B	~158.6	NA	IncFII	MOBF	Type F	*virb4, MOBB, t4cp1 traF, traE, traB, traL, trbC, traK, traV, traW, traU*
	~7	PTU-E3	Col440I	MOBP	-	-

COPLA: https://castillo.dicom.unican.es/copla
[a]PTU: plasmid taxonomic unit; NA: PTU not assigned
[b]Inc groups: Incompatibility plasmid groups based on genes for replication
[c]MOB: plasmid mobility classes based on genes for conjugation
[d]MPF: mating-pair formation classes based on genes for secretion of DNA from donor cell to recipient cell
- Not found by COPLA classifier
Results based on Nanopore-polished and Illumina-hybrid assemblies
